# Developing a transdiagnostic Ecological Momentary Assessment protocol for psychopathology

**DOI:** 10.1002/mpr.2028

**Published:** 2024-07-19

**Authors:** Alberto Jover Martínez, Lotte H. J. M. Lemmens, Eiko I. Fried, Anne Roefs

**Affiliations:** ^1^ Clinical Psychological Science Faculty of Psychology and Neuroscience Maastricht University Maastricht The Netherlands; ^2^ Clinical Psychology Faculty of Social and Behavioral Sciences Leiden University Leiden The Netherlands

**Keywords:** Ecological Momentary Assessment (EMA), measurement, network approach, psychopathology, transdiagnostic

## Abstract

**Objectives:**

The network approach to psychopathology posits that mental disorders emerge from dynamic interactions among psychopathology‐relevant variables. Ecological Momentary Assessment (EMA) is frequently used to assess these variables in daily life. Considering the transdiagnostic nature of the network approach to psychopathology, this study describes the development of a transdiagnostic EMA protocol for psychopathology.

**Methods:**

First, 96 clinicians completed an online survey, providing three EMA constructs for up to three disorders they specialize in, and three EMA constructs relevant across disorders (transdiagnostic constructs). Second, 12 focus groups were conducted with clinical experts for specific types of diagnoses (e.g., mood disorders, anxiety disorders). Finally, a selection of items was reached by consensus. Two raters independently coded the online survey responses with an inter‐rater agreement of 87.3%.

**Results:**

Jaccard indices showed up to 52.6% overlap in EMA items across types of diagnoses. The most frequently reported transdiagnostic constructs were mood, sleep quality, and stress. A final set of EMA items is created based on items' frequency and informativeness, ensuring completeness across diagnoses and minimizing burden.

**Conclusions:**

The described procedure resulted in a feasible EMA protocol to examine psychopathology transdiagnostically. Feasibility was helped by the overlap in mentioned symptoms across disorders. Such overlap raises questions about the validity of DSM categories.

## 1 INTRODUCTION

The medical model has been the basis of research and treatment in clinical psychology and psychiatry for decades (Cooper, 1995). This model states that a mental disorder's symptoms are caused by an underlying common cause, typically of biological origin (e.g., genetics, chemical imbalance in the brain; Deacon, 2013; Scull, 2021). The default for diagnosing mental disorders is a classification provided in the Diagnostic and Statistical Manual of Mental Disorders (5th ed.; DSM5; American Psychiatric Association, 2013), which is based on this medical model and has been criticized for its lack of validity (Borsboom, 2008; Fried, 2017; Fried et al., 2020; Regier et al., 2013; Santor et al., 2006) and clinical utility (Holmes et al., 2018; Layard & Clark, 2015; Ruggero et al., 2019).

The network approach to psychopathology has provided an alternative to the medical model positing that the dynamic interplay of symptoms constitutes mental disorders (Borsboom, 2017). Moreover, the network approach is not limited to symptoms of disorders, but includes other relevant variables, such as certain cognitions, behaviors, and social circumstances (Roefs et al., 2022). Different methodologies have been developed over the years to test this approach using different types of data (Borsboom et al., 2021). A frequently used method to test the network approach uses cross‐sectional data in combination with, for example, an Ising model, a Gaussian graphical model, or a mixed graphical model (Borsboom et al., 2021). These analyses result in a “cross‐sectional” network, which usually represents individual differences (Borsboom et al., 2021; for examples see Fried et al., 2018; Hoffart et al., 2021, or van Borkulo et al., 2015). Cross‐sectional networks can be useful as exploratory tools (von Klipstein et al., 2021). However, due to the lack of temporal data, such networks do not provide information about the dynamic interplay within a person that the network approach proposes as a mechanistic explanation of psychopathology. The goal of this study is to develop a measurement protocol that permits the acquisition of temporal data within individuals with an appropriate time‐resolution, to optimally test predictions of the network approach to psychopathology.

To study the dynamic interplay between elements that the network approach proposes, we need to consider how the network elements develop over time *within* an individual (Molenaar, 2004). That is why studies interested in time dynamics use time series analysis, such as the vector autoregressive (VAR) model (Zivot & Wang, 2006), or one of its extensions such as the multi‐level VAR (mlVAR; Bringmann et al., 2013). The VAR model provides information about the relationships of each variable at time point *t* with itself and all other variables at time point *t *−1 for each individual. The mlVAR model adds a random effects component that distinguishes between‐ and within‐individuals variance. Such models require intensive longitudinal data, as can be collected with Ecological Momentary Assessment (EMA; Shiffman et al., 2008).

EMA studies consist of repeatedly asking participants in‐the‐moment questions about phenomena relevant for a study (e.g., behaviors, thoughts, experiences, emotions) during a period of time (Shiffman et al., 2008). EMA has several advantages over laboratory and retrospective survey studies. First, EMA permits the study of time dynamics, which feature centrally in the network approach to psychopathology. Second, EMA decreases the risk of recall bias because the assessments concern in‐the‐moment experiences. Third, EMA is more ecologically valid because the answers are given within the context of daily life. Therefore, EMA is a valuable addition to the toolkit researchers can use to study psychopathology (Russell & Gajos, 2020; Smyth & Stone, 2003; Wenze & Miller, 2010).

Most EMA studies on the network approach have focused on a single disorder, mostly Major Depressive Disorder (MDD; Wichers et al., 2021). Nevertheless, focusing on just one disorder is likely suboptimal because half of the people with a mental disorder receive two or more diagnoses (Kessler et al., 2005; Kim & Eaton, 2015; Lilienfeld, 2014; Nolen‐Hoeksema & Watkins, 2011; Sauer‐Zavala et al., 2017). According to the network approach, comorbidity occurs because similar symptoms can occur in multiple disorders. The onset of such symptoms increases the likelihood of activating other symptoms, belonging to different disorders as well (comorbidity hypothesis, Cramer et al., 2010). Therefore, it is important to investigate the network approach to psychopathology transdiagnostically, instead of only focusing on single disorders. The current study entails the development of such a transdiagnostic EMA measurement protocol of psychopathology.

To be optimally valuable, an EMA measurement protocol needs to be carefully designed (e.g., number of surveys per day, number of items per survey, schedules of the surveys, randomization of surveys, length of study, etc. need to be considered; Wright & Zimmermann, 2019). In many studies, EMA protocols use items from questionnaires and surveys that are developed for laboratory or retrospective survey studies (Wichers et al., 2021). Many of those questionnaires are designed to capture either a certain diagnostic category (e.g., anxiety disorders) or a certain transdiagnostic construct (e.g., insomnia). These types of questionnaires do not align well with the network approach, because (1) the focus is often on one diagnostic category or one transdiagnostic construct, (2) questionnaires are often long, and (3) the questions are often framed retrospectively, ask participants to consider a certain past period. Therefore, an EMA measurement protocol for transdiagnostic assessment in daily life and for network modeling purposes is needed (Wichers et al., 2021).

In the current study, we aim to develop a dedicated transdiagnostic EMA protocol for psychopathology. This protocol includes symptoms of mental disorders as well as other psychopathology‐relevant variables, such as social context. Moreover, the protocol includes variables that are relevant for multiple disorders (i.e., transdiagnostic variables). In addition to such transdiagnostic variables, we also aim to incorporate disorder‐specific variables that are crucial for specific disorders to ensure the comprehensiveness of the protocol. Developing a measurement protocol encompassing such a large range of variables faces a number of challenges. First, the tradeoff between information gain for researchers and burden for participants. On the one hand, the number of items per measurement moment needs to be limited to mitigate participant burden and promote compliance and careful answering of questions (Eisele et al., 2020). On the other hand, transdiagnostic measurement calls for a broad range of constructs (Eisele et al., 2020). Therefore, it is crucial to carefully select a limited set of items that accurately and optimally capture the phenomena of interest. Second, in EMA studies one needs to consider how dynamic the variables of interest are, that is, the expected fluctuation throughout the day (e.g., it makes little sense to query about sleep quality more than once per day).

These challenges are tackled by leveraging two sources of information. First, an online survey of clinicians is deployed to determine the most informative variables for every disorder. Second, we conduct focus groups with clinical experts of specific disorders. Focus groups are chosen as a complementary, high quality and reliable sources of information to the survey of clinicians. Clinical experts' level of experience over the years renders them a reliable and effective source of information (Rauf et al., 2014; Willis et al., 2009). The EMA protocol is developed following three steps: (1) determining the most relevant items in the online survey, (2) complementing the information from the survey with the information from the focus groups, and (3) coding all information, computing interrater reliability, and carefully selecting a final list of items. The whole process is thoroughly described in the present paper, and the resulting EMA measurement protocol is presented.

## 2 METHOD

### 2.1 Overview

The current study consisted of three steps: (1) an online survey administered to clinicians, (2) focus groups with clinicians of 12 categories of mental disorders, and (3) combining the information gathered from those two sources.

### 2.2 Participants

#### 2.2.1 Online survey of clinicians

Our international recruitment targeted officially licensed mental health clinicians (according to own country guidelines), holding at least a master's degree in clinical psychology, psychiatry, or a similar mental health care specialization. Participants were recruited via advertisements distributed among various psychotherapy associations from the Netherlands, the USA, and Europe, and via social media and networks of members of our research team. The survey was approved by the ethical committee of the Faculty of Psychology and Neuroscience of Maastricht University, and was pre‐registered on AsPredicted (#62825; https://aspredicted.org/79X_XY5). Of 241 potential participants who clicked on the survey's link, 96 (39.83%) completed it. Participants characteristics can be found in Table 1.

**TABLE 1:** Work‐related demographic characteristics of clinicians participating in the survey study (*n* = 95).

Characteristic	*n*	%
Position		
Behavioral Scientist	2	2.1
Licensed health psychologist	32	33.7
Licensed child‐ and youth psychologist	5	5.3
Licensed psychotherapist	15	15.8
Licensed clinical (neuro)psychologist	23	24.2
Psychiatrist	5	5.3
Other	13	13.7
Recruitment source		
A psychotherapy association	57	60.0
LinkedIn	33	34.7
Social media	5	5.3
Country		
Belgium	9	9.5
Germany	4	4.2
Greece	1	1.1
Iceland	1	1.1
Netherlands	77	81.1
New Zealand	1	1.1
Peru	1	1.1
United States of America	1	1.1
Work setting^a^		
General hospital	8	8.4
Mental health care center	64	67.4
Private practice	24	25.3
Other	8	8.4
Type of care^a^		
Inpatient	22	23.2
Outpatient	86	90.5
PhD		
Yes	32	33.7
No	63	66.3
Stream of thought^a^		
Cognitive Behavioral Therapy	70	73.7
Psychoanalytic and/or psychodynamic	16	16.8
EMDR	37	38.9
Family systems	13	13.7
Humanistic	9	9.5
Dialectical behavioral	7	7.4
Interpersonal	15	15.8
Integrative	16	16.8
Emotion focused	15	15.8
Narrative	4	4.2
Motivational interviewing	10	10.2
Other	12	12.6
Main type of patient		
Children and/or adolescents	6	6.3
Adults	82	86.3
Elderly	6	6.3
Families	1	1.1
Work experience		
1–5 years	11	11.6
6–10 years	18	18.9
11–15 years	20	21.1
16–20 years	15	15.8
21–25 years	14	14.7
26–30 years	4	4.2
More than 30 years	13	13.7
Working hours		
1 day or less per week	4	4.2
Between 2 and 4 days per week	60	63.2
Full time (5 days per week)	31	32.6

*Note*: *n* = 95 for demographic information because one participant did not answer the demographic questions.

^a^ Multiple answers possible.

#### 2.2.2 Focus groups

Experts were selected through purposive sampling, as is often done in focus group research (Ryan et al., 2014), based on their contribution to their respective clinical and research fields, and were recruited via e‐mail. In total, 12 focus groups were carried out, one per disorder group. Most focus groups consisted of 3 experts: mood disorders, anxiety disorders, substance use disorders, eating disorders, somatoform disorders, psychotic disorders, neurodevelopmental disorders, and sexual disorders. The trauma and stressor related disorders, personality disorders, sleep disorders, and conduct disorders focus groups consisted of 2 experts. The focus groups were pre‐registered and approved by the faculty's ethical committee (AsPredicted #62825; https://aspredicted.org/79X_XY5).

### 2.3 Design

#### 2.3.1 Online survey of clinicians

##### Contents of survey

The survey was administered via the online platform Qualtrics (https://www.qualtrics.com) and inquired about the types of items that clinicians would include for an EMA study investigating the entire range of psychopathology (Roefs et al., 2022). Participants named the three EMA items they considered most relevant for the (up to three) disorders they specialize in. Afterward, they named 3 EMA items that they would ask people with any disorder (i.e., transdiagnostic items).

##### Analyses questionnaire

All provided answers (*k* = 1004) were coded by two independent raters (R1 and R2) and coded to denote the construct the item taps into. For example, both items “Do you feel an urge or a craving to consume drugs right now?” and “Do you have a huge desire to take drugs?” were coded to reflect the same construct “craving.” We calculated inter‐rater reliability, and afterward, any disagreements were resolved together with a third rater (R3). Finally, the frequencies of the constructs were explored to determine the more popular ones. The amount of overlap between the different mental disorders' constructs was calculated by means of Jaccard similarity indices. This index ranges between 0 and 1, with higher values indicating more similarity. It is operationalized as the length of the union divided by the size of the intersection between the sets: Jaccard Similarity = (number of observations in both sets)/(number in either set). This operationalization is written in notation form as J(A, B) = |A∩B|/|A∪B|.

#### 2.3.2 Focus groups

To ensure that no relevant items were overlooked, we utilized focus groups with clinical experts to gather richer information about relevant items in psychopathology EMA studies. Each focus group consisted of a 90‐min semi‐structured interview and was carried out and recorded via zoom (https://zoom.us). The experts were rewarded with a 15 euros voucher.

##### Semi‐structured interview protocol

Each focus group concerned a specific type of mental disorder, making sure that the entire range of psychopathology was covered. There were two parts in each focus group: The first part was concerned with etiology, clinically relevant factors, and theory, and the second part was about the items they would include in the EMA protocol. Experts were also encouraged to think thoroughly if the items should be asked momentarily (i.e., several times per day), daily, or weekly, to ensure that items can be phrased according to how they are thought to fluctuate over time.

#### 2.3.3 Putting it all together

The information obtained from the online survey was integrated with the information obtained in the focus groups. First, the most frequently mentioned constructs in the online survey per disorder were selected if such constructs were suitable and informative for a transdiagnostic EMA study. Selection was based on two criteria: (1) sufficient within‐person variance was to be expected. For example, an individual's attachment style does not vary frequently enough to warrant multiple assessments during the day. (2) The questions needed to be about concepts that are understood easily by participants. We excluded too complex or abstract concepts, such as the adequacy of an emotional response given a certain situation, and how spiritually fulfilling an activity feels.

Second, the list of constructs obtained from the online survey was complemented with constructs from the focus groups we deemed important from a content perspective while still considering the criteria mentioned above. Third, the number of constructs was reduced to limit participant burden. To do so, R3 and R1 each made a selection of constructs based on two criteria: (1) constructs that were more transdiagnostic (i.e., that were mentioned for more disorders) were favored, (2) constructs that were central for specific disorders were favored (e.g., compulsions may only be relevant for Obsessive Compulsive Disorder rather than the entire group of anxiety disorders, but it is extremely central for this disorder). After the selections were made, there was a discussion to solve the disagreements on the selections. Finally, the list was reduced based on the overall relevance of the constructs to keep the list as short as possible. For each selected construct, an adequately phrased item was formulated, if possible, based on the ESM repository (Kirtley et al., 2019).

## 3 RESULTS

### 3.1 Step 1: Online survey of clinicians

Table 2 shows a summary of which disorders were most frequently treated by the participating clinicians. Participating clinicians could choose up to three disorders, and they were asked to mention the disorders they treat in treatment‐frequency order (i.e., 1st selected disorder is most frequently treated). For determining the underlying constructs (e.g., craving alcohol) that items (e.g., “intense desire to consume alcohol”) listed by clinicians tapped into, the inter‐rater agreement was 87.3%. Disagreements between R1 and R2 were resolved by R3. The 5 most frequently mentioned constructs per type of disorder, and the transdiagnostic constructs are summarized in Table 3.

**TABLE 2:** Information on the mental disorders participating clinicians treated.

Category of disorder	1st disorder		2nd disorder		3rd disorder		In total	
	*n*	%	*n*	%	*n*	%	*n*	%
Anxiety disorders and OCD	13	13.5	7	8.8	16	24.2	36	14.9
Disruptive, impulse‐control, and conduct disorders	1	1.0	2	2.5	1	1.5	4	1.7
Eating disorder	2	2.1	1	1.3	1	1.5	4	1.7
Mood disorders	24	25.0	19	23.8	14	21.2	57	23.6
Neurodevelopmental disorders	4	4.2	4	5.0	1	1.5	9	3.7
Personality disorders	24	25.0	14	17.5	8	12.1	46	19.0
Psychotic disorders	6	6.3	4	5.0	5	7.6	15	6.2
Sexual disorders	0	0.0	1	1.3	0	0.0	1	0.4
Somatoform disorders	1	1.0	1	1.3	2	3.0	4	1.7
Sleep disorders	1	1.0	3	3.8	2	3.0	11	4.5
Substance use/addiction disorders	6	6.3	3	3.8	2	3.0	11	4.5
Trauma and stressor disorders	14	14.6	21	26.3	15	22.7	50	20.7
Number of responses	96	100	80	100	66	100	242	100

*Note*: *n* = 96. Participants could mention up to three main categories of disorders they mostly work with. This table displays the percentage of participants that chose each disorder in each position.

**TABLE 3:** Rank order of the most commonly mentioned constructs for the disorder‐specific categories.

Category	1st construct	2nd construct	3rd construct	4th construct	5th construct
Mood disorders	Mood	Energy	Enjoyment	Activity	Suicidal ideation
Anxiety disorders (and OCD)	Anxiety	Avoidance	Coping	Tension	Activity, etc.*
Trauma and stress related disorders	Avoidance	Feeling safe	Flashbacks*	Stress*	Coping
Substance abuse disorders	Urge/craving	Substance use	Happiness*	Interpersonal support*	Used amount*
Somatoform disorders	Ability to move	Ability to relax*	Context*	Functioning*	Mood, etc.*
Eating disorders	Body‐image*	Compensatory behaviors*	Eating mood*	Self‐control*	Self‐esteem, etc.*
Psychotic disorders	Anxiety	Paranoid	Auditive hallucinations	Mood	Burden, etc.*
Neurodevelopmental disorders	Concentration*	Mood*	Overstimulation*	Sleep quality*	Alertness, etc.**
Personality disorders	Interpersonal problems*	Interpersonal satisfaction*	Emotion regulation**	Stress**	Interpersonal connectedness, etc. (mood)***
Sleep disorders	Sleep quality	Feeling rested	Tension*	Energy*	Falling asleep, etc.**
Sexual disorders	Interpersonal sexual	Masturbation	Sexual pain	‐	‐
Disruptive, impulse‐control, and conduct disorders	Anger	Anger management*	Coping strategy*	Fights*	Frustration tolerance, etc.*
All disorders	Mood	Anxiety	Sleep quality	Avoidance	Coping
Transdiagnostic constructs	Mood	Sleep quality	Stress	Anxiety*	Coping*

*Note*: Frequencies were collapsed across disorders, and in the last row across transdiagnostic constructs. The asterisks in this table represent ties. For example, if several constructs are followed by an asterisk “*” those constructs were mentioned the same number of times. Only constructs with the same number of asterisks are tied together.

**FIGURE 1:**
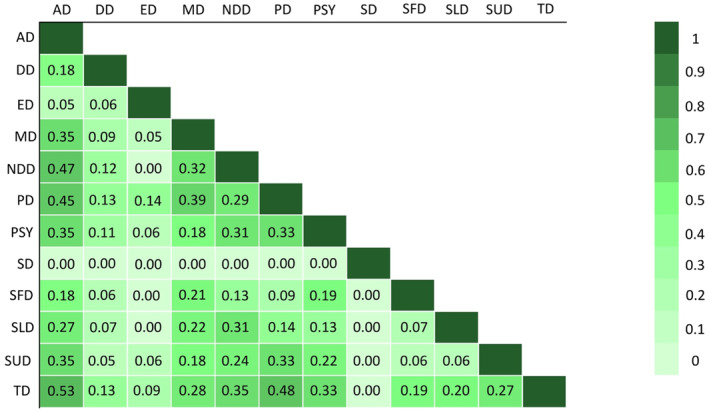
Heatmap Jaccard Similarity Indices for all Categories of Disorders. This heatmap shows the amount of overlap between disorders. An index of 1 means total overlap, and an index of 0 means total difference. AD, anxiety disorders; DD, disruptive, impulse‐control, and conduct disorders; ED, eating disorders; MD, mood disorders; NDD, neurodevelopmental disorders; PD, personality disorders; PSY, psychotic disorders; SD, sexual disorders; SFD, somatoform disorders; SLD, sleep disorders; SUD, substance use/addiction disorders; TD, trauma and stress related disorders.

The largest Jaccard similarity index was observed between the items mentioned for anxiety disorders and trauma and stress related disorders (0.53), followed by personality disorders and trauma and stress related disorders (0.48), and anxiety disorders and neurodevelopmental disorders (0.47). The heatmap in Figure 1 shows the overlap across all disorders.

**FIGURE 2:**
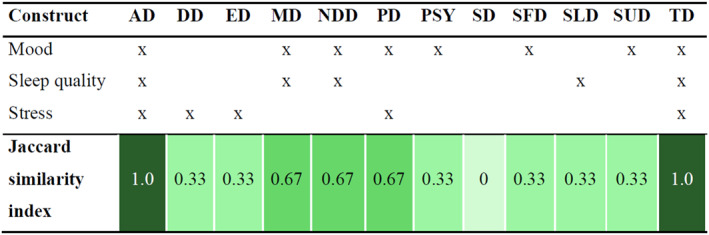
Overlap between transdiagnostic constructs mentioned and disorder‐specific constructs. AD, anxiety disorders; DD, disruptive, impulse‐control, and conduct disorders; ED, eating disorders; MD, mood disorders; NDD, neurodevelopmental disorders; PD, personality disorders; PSY, psychotic disorders; SD, sexual disorders; SFD, somatoform disorders; SLD, sleep disorders; SUD, substance use/addiction disorders; TD, trauma and stressor related disorders.

The degree of overlap between the disorder‐specific constructs and the transdiagnostic constructs was investigated by means of Jaccard similarity indices as well. The top 3 most mentioned transdiagnostic constructs were mood, sleep quality, and stress. As Figure 2 displays, at least one transdiagnostic construct was also mentioned as a disorder‐specific constructs for all disorders, except for sexual disorders. All three transdiagnostic constructs were mentioned for anxiety disorders, and trauma and stressor‐related disorders.

### 3.2 Step 2: Focus groups

Appendix A shows an overview of the constructs proposed by the focus groups divided in the momentary questions, daily questions, and weekly constructs. Most suggested constructs varied throughout the day, and overlapped considerably across focus groups. For example, mood was mentioned in six focus groups, and stress as well as avoidance in four. These constructs, as well as other constructs that varied throughout the day, were included in the final questionnaire. Some focus groups, such as the focus groups for sleep disorders and personality disorders, did not mention many constructs that varied throughout the day. In these focus groups, it was considered that constructs that varied more slowly (e.g., that varied from day to day, or from week to week) were more relevant for that type of psychopathology. Of these daily and weekly constructs, there was some overlap across focus groups. For example, sleep was mentioned in eight focus groups, substance use in three, and medication in two. These constructs were included as daily constructs in the final questionnaire. The fewest constructs were gathered for the weekly constructs, for which only suicidality was mentioned sufficiently commonly to warrant inclusion.

### 3.3 Step 3: Putting it all together

There was considerable overlap between the online survey and the focus groups. However, some constructs were only mentioned in the focus groups. Some of these constructs were included in the EMA protocol due to their relevance. For example, feeling lonely, loss of control, and life meaning were constructs that were only mentioned in the focus groups, and were included in the EMA protocol.

The resulting EMA questionnaire includes a maximum of 35 momentary items, 26 daily items, and 4 weekly items, which can be found in Appendix B. Some surveys may include fewer items due to conditional branching (e.g., if participants state that they are alone, they will not be asked who they are with).

All included questions were phrased on 7‐point Likert scales. A 7‐point Likert scale is used because it is within the optimal range of options (Simms et al., 2019), to permit participants to select a middle point (i.e., answering option 4 on the 7‐point scale), and to enable sufficiently fine‐grained answering. Likert scales were used rather than Visual Analogs Scales (VAS) because they are faster to answer, and easier to use for younger and older people, and populations with a lower education level (Fryer & Nakao, 2020; Trimmel & Trimmel, 2017). Moreover, quantification of answers is easier for Likert scales (Trimmel & Trimmel, 2017), and psychometric properties are comparable to VAS (Simms et al., 2019).

## 4 DISCUSSION

In the present study, a comprehensive transdiagnostic EMA protocol for psychopathology was developed based on an online survey completed by clinicians and focus groups with clinical experts. The degree of overlap across disorders, as well as between disorder‐specific and transdiagnostic constructs was explored. The degree of overlap was substantial between disorders, including the transdiagnostic constructs. This overlap helped in keeping the final set of EMA items within acceptable limits for participants and underlines the rationale for studying psychopathology transdiagnostically. The study resulted in an EMA measurement protocol consisting of up to 35 momentary items, up to 26 daily items, and 4 weekly items (some items may not be answered due to conditional branching).

As expected from a transdiagnostic approach, the degree of overlap across the different mental disorders was substantial. In some cases, almost half of the disorder‐specific answers overlapped between two disorders, and in one case—between anxiety disorders and trauma and stress related disorders—the overlap was above 50%. Similarly, the transdiagnostic constructs overlapped considerably with the disorder‐specific constructs. Only for sexual disorders, no overlap was found with the top‐3 transdiagnostic constructs. Note that a limitation of the present study is that for some disorders only few responses (i.e., fewer than 10) were obtained in the online survey, because we assessed a limited number of clinicians treating such disorders. However, this lack of responses was compensated by the information obtained in the focus groups. Other limitations of the online survey are the overrepresentation of some nationalities (i.e., Dutch clinicians) and that the patients' perspective was not considered. Future research should address these limitations.

The large overlap across disorders makes sense considering the overlap of criteria between DSM categories (Forbes et al., 2023). Such overlap between diagnoses casts doubts on the validity of the DSM categories because they do not seem to be well delineated. Promising alternative classifications of psychopathology, such as the Hierarchical Taxonomy of Psychopathology (Ruggero et al., 2019), and the Research Domain Criteria (RDoC; Insel et al., 2010) project also attempt to circumvent these problems of the DSM classification. The network approach goes one step further, as it takes an idiographic perspective to psychopathology. Moreover, the network approach shares a vision on comorbidity with Hi‐TOP and RDoC that aligns with the present results: “There are also symptoms that do not clearly belong to one or the other disorder, because they receive and send out effects to the symptoms in both of the disorders (i.e., overlapping symptoms) […] which we propose to call a bridge symptom. We hypothesize that in clinical practice, such bridge symptoms turn up as symptoms that are used in diagnostic schemes, such as the DSM‐IV, for multiple disorders.” (Cramer, et al., 2010, p. 140). Therefore, comorbidity is not due to a (bi)directional relationship between two latent factors (i.e., disorders). Instead, it is due to the effects that spread out from bridge symptoms. The observed overlap of constructs between disorders suggests that there could be plenty of such bridge symptoms.

A number of studies have investigated comorbidity from a network approach. Most of these studies were cross‐sectional and focused on comorbidity between MDD and symptoms of other disorders, such as generalized anxiety disorder, post‐traumatic stress disorder, bulimia nervosa, substance use disorder, or bipolar disorder. Some studies observed that symptoms of those disorders often co‐occur (Afzali et al., 2017; Heeren et al., 2018; Jones et al., 2018; Lazarov et al., 2020; Shim et al., 2020), whereas others do not (de Haan et al., 2020; Rogers et al., 2019). The studies suggest some methodological explanations about why not bridge symptoms were found, like large number of nodes in the network model, considerable quantity of missing data for many items, and the methodological challenges associated with imputing missing values in network analyses. However, another possibility suggested is that bridge symptoms do not have time to manifest in fast episodes of a disorder. Instead, they may manifest during more chronic episodes when there is time for such symptoms to unfold.

A recent review of network studies only found two studies who use temporal data to study comorbidity (Wichers et al., 2021). For these studies, the results are also mixed: one study found large individual differences in bridge symptoms (Kaiser & Laireiter, 2018), whereas the other did not find any evidence thereof (Groen et al., 2020). The authors mention that their study design did not allow for the investigation of bridge symptoms during all phases of psychopathology development. They suggest that bridge mental states may be more relevant during the period when comorbidity first develops or re‐develops. For this reason, it is crucial to develop ways to determine when is the best moment to assess variables. Future research should explore whether triggers based on passive data or warning signals (e.g., a threshold score) can be used to signal when a variable can best be assessed.

The EMA protocol resulting from this study opens the possibility to study psychopathology from a transdiagnostic approach in daily life. Developing a measurement protocol like this is necessarily partly a subjective process. This study attempted to reduce the degree of subjectivity as much as possible and to include the most clinically relevant items. Several design strengths contributed to these goals. First, expert clinicians were asked for input, guaranteeing a close link with clinical practice. Second, participants and experts were explicitly asked to mention variables that were expected to fluctuate throughout the day, making them suitable for inclusion in an EMA protocol. Third, all ratings were conducted independently by at least two researchers, and any disagreements were settled in consultation with a third researcher, reducing subjectivity.

Notable as well is that the time scale was adapted to the type of variable that was assessed: momentary items (8 times per day), morning items, evening items, and weekly items, based on the expected degree of fluctuation. For example, it makes sense to ask about mood several times a day, whereas it suffices to assess sleep quality only in the morning. Therefore, psychological phenomena must be asked at the proper time scale to properly capture fluctuations and to reduce participant burden. Some researchers have already highlighted the relevance of distinguishing the time scales of different psychological phenomena (Wichers et al., 2021). Specifically, research suggests that different psychological phenomena exert their influence at a different level (Wichers et al., 2021). Specifically, the dynamics between micro‐level momentary affective states are actually the building blocks for the development or maintenance macro‐level symptoms. This difference requires different methodological considerations such as a different measurement frequency.

Unfortunately, network estimation methods are not able to deal with variables measured at a different time scales yet, despite how central that is for the network approach to psychopathology. Therefore, it is crucial to develop methods to determine variables' optimal time scale, and methods to combine variables measured at different time scales. A possible way to determine a variable's optimal time scale might be fitting autoregressive models with longer lags to see which lag is more predictive. Variables which variance is better accounted for with longer lags might be better captured with longer time scales. Regarding possible ways of modeling variables measured at different frequencies,multi‐layered networks might be a possibility. With this approach, variables measured at the same frequency could be used to build a network, which would lead to a network per assessment frequency. Next, the networks for each assessment frequency can be integrated in a multi‐layer network (Blanken et al., 2021).

Taken together, the results of this study suggest that a transdiagnostic approach to psychopathology, and its study in daily life within individuals, is a promising direction of research, which can now be explored in EMA studies. Moreover, this approach fits well with the network approach to psychopathology. The usefulness of this questionnaire extends beyond the study of the network approach to psychopathology in daily life. The time‐series data that can be obtained with this protocol can be analyzed in different ways from different perspectives. Data will need to be collected to test if there is enough within‐subjects variance for each item, if the present questionnaire is related to standardized measures of psychopathology, and to determine if any changes regarding the measurement frequency are needed (Schreuder et al., 2020). If items are asked at a time scale that is not frequent enough, the variations will not be captured, and if they are asked too frequently, the participant burden is unnecessarily high. Moreover, the validity and reliability of the EMA protocol needs to be studied as well. The EMA protocol can then be further refined for future studies.

## AUTHOR CONTRIBUTIONS

**Alberto Jover Martínez**: Conceptualization; methodology; formal analysis; investigation; data curation; writing—original draft; visualization; project administration. **Lotte H. J. M. Lemmens**: Conceptualization; writing—review and editing; methodology. **Eiko I. Fried**: Conceptualization; methodology; writing—review and editing; funding acquisition. **Anne Roefs**: Conceptualization; methodology; formal analysis; writing—review and editing; supervision; project administration; funding acquisition.

## CONFLICT OF INTEREST STATEMENT

The authors declare no conflicts of interest.

## ETHICS STATEMENT

The survey was approved by the ethical committee of the Faculty of Psychology and Neuroscience of Maastricht University, and was pre‐registered on AsPredicted (#62825; https://aspredicted.org/79X_XY5).

## PATIENT CONSENT STATEMENT

No patients participated in this study.

## PERMISSION TO REPRODUCE MATERIAL FROM OTHER SOURCES

No materials were reproduced from other sources.

## CLINICAL TRIAL REGISTRATION

This study was not a clinical trial.

## Supporting information

Supplementary Material

## Data Availability

The data that support the findings of this study are available on request from the corresponding author. The data are not publicly available due to privacy or ethical restrictions.
