# Supplementary material for: Developing a transdiagnostic Ecological Momentary Assessment protocol for psychopathology
Source: Int J Methods Psychiatr Res. 2024 Jul 19;33(3):e2028. doi: 10.1002/mpr.2028 (PMC11258473; doi:10.1002/mpr.2028)
Supplement: Supplementary file 1 — Supplementary Material [file MPR-33-e2028-s001.docx]

**Appendix A**

| Appendix A1 Proposed momentary constructs by the focus groups | | | | | | | | | | | |
| --- | --- | --- | --- | --- | --- | --- | --- | --- | --- | --- | --- |
| MD | AD | ED | TD | SUB | SFD | PSY | SD | SLD | PD | DD | NDD |
| Other Activity | Tension | Feelings of control | Intrusions | Substance: type, amount, frequency | Somatic symptoms | Hallucinations | Sexual desire | Sleepiness | Coping styles | Anger/Annoyed/ irritated/frustrated | Restlessness |
| Mood | Other Activity | Concerns | Avoidance | Craving | Worry about symptoms | Suspiciousness | Sexual arousal | Alertness | How are you doing? | Revenge-plotting | What are you doing wrong? |
| Positive Affect | Avoidance | Negative feelings about body | Hyperarousal/startle response | Anxiety | Stress | Delusions | Object of arousal | Mood | Levels of personality scale | Thinking of bad things to do | Forgetfulness |
| Anhedonia | Mood | Preoccupation about food and eating | Detachment | Mood | Avoidance/ coping | Hyperarousal | Fantasizing |  |  | Guilt | Distractibility |
| Motivation | Sadness | Compensatory behaviours | Concentration | Daily activities | What do you think that causes your complaints | Loss of control | Sexual activity |  |  | Do you want to annoy people? | Social interaction |
| Restlessness | Happiness | Avoidance of seeing the body | Coping style | Do people around you use | Preoccupation (body check, thinking about complaints) | Preoccupation | Feelings about sexual activity |  |  | Verbal/physical aggression | Mood/mood swings |
| Tension | Energetic | Body checking |  | Are you intoxicated | Overload | Social experiences | Level of stress |  |  | Did you do something bad without thinking | Menstruation cycle |
| Anxiety | Tiredness | Self-criticism |  | Context | Pain coping behaviours | People around | Relationship satisfaction |  |  | Self-control | Hormonal contraception |
| Nervousness | Bodily symptoms | Eating pattern |  | Stress | Limitations in functioning as a consequence of symptoms | anhedonia | Perceived partner responsiveness |  |  | Resentment, mistrust, cynicism | Social support |
| Rumination | Context | Interpersonal relations |  | Boredom |  | Reliving experiences | Time spent with partner |  |  | Perceived wrongdoing | Task-focus |
| Activity | Mulling | Behaviour in life |  |  |  | Mood | Level of interaction |  |  | Guiltiness | Irritability |
|  |  |  |  |  |  |  |  |  |  |  | Focus/concentration |
|  |  |  |  |  |  |  |  |  |  |  | Fatigue |

| Appendix A2 Proposed daily items by the focus groups. | | | | | | | | | | | |
| --- | --- | --- | --- | --- | --- | --- | --- | --- | --- | --- | --- |
| MD | AD | ED | TD | SUB | SFD | PSY | SD | SLD | PD | DD | NDD |
| Sleep | Panic attack | Sleep | Intrusions | Sleep quality | Sleep | Sleep | Sexual problems | How did you sleep? | Daily quality of life | Too angry /easily annoyed | Sleep |
| Substance use | avoidance | How was your day | Detachment | Amount of drinks | Resting | Emotion regulation | Catastrophizing | How much did you sleep? | Interpersonal tension | Substance use |  |
| Perspective/ hope | Time spent in compulsions | Binges |  | Were you intoxicated when filling out the surveys? | Physical/ mental/ social limitations |  | Relationship conflicts | Physically tense | Interpersonal stressful events |  | Medication |
| Self-esteem | Time spent worrying | Restrain |  | Drug usage | Activity levels |  | Coping with relationship conflict | Mentally alert | Demoralisation |  | Compliance to medication |
| Self-confidence | Sleep | Self-harm |  | Meaningful activity | Medication use |  | Initiative to have sex | Daily functioning |  |  | Eating pattern |
| Enjoyment (food) | Suicidal ideation | (bad) memories |  | Physical wellbeing | Acceptance |  |  |  |  |  | Functioning |
|  | coping | Compensation behaviors |  |  | Information seeking on internet |  |  |  |  |  | How ordered is your house today? |
|  | Is there someone I can talk to? | Body checking behaviors |  |  |  |  |  |  |  |  | Social interactions |
|  | Meaning in life | Avoidance |  |  |  |  |  |  |  |  | Social media |
|  | Satisfaction in life |  |  |  |  |  |  |  |  |  | How was your day? |
|  | Purpose in life |  |  |  |  |  |  |  |  |  |  |
|  | How was your day |  |  |  |  |  |  |  |  |  |  |

| Appendix A3 Proposed weekly constructs by the focus groups | | | | | | | | | | | |
| --- | --- | --- | --- | --- | --- | --- | --- | --- | --- | --- | --- |
| MD | AD | ED | TD | SUB | SFD | PSY | SD | SLD | PD | DD | NDD |
| Enjoyment (music) | Social support | Suicidality | Intrusions | Positive/ negative life events | Medical investigation |  |  |  | Personality functioning questionnaire | Did you get into a physical/verbal fight? | Menstruation cycle |
| Suicidal thoughts |  |  | Avoidance | Quit smoking |  |  |  |  | suicidality |  |  |
|  |  |  | Hyperarousal | Therapy sessions |  |  |  |  | self-harm |  |  |
|  |  |  | Startling |  |  |  |  |  |  |  |  |
|  |  |  | PCL/PSS |  |  |  |  |  |  |  |  |

**Appendix B**

All items, unless otherwise noted, are answered on vertical 7-point Likert scales with the following anchors: Not at all, 2, 3, 4, 5, 6, Extremely.

Appendix B1 Momentary items.

Please indicate to what extent you feel the following mood states and physical sensations at this moment:

- Sad
- Guilty
- Happy
- Hopeless
- Anxious
- Stressed
- Overwhelmed
- Angry
- Calm
- Energetic
- Lonely
- Paranoid
- In pain
- Dizzy
- Nauseous
- Trembling
- Like my heart is racing

Please indicate to what extent you agree with the following statements:

- At this very moment, I look forward to completing the activities that I planned for later
- At this very moment, I am satisfied with myself
- At this very moment, I am satisfied with my physical appearance
- At this very moment, I experience cravings

If the question “At this very moment, I experience cravings” is answered with anything other than “Not at all”. The following question is triggered:

- What do you crave? (You can select more than one answer.)

With the following answer options: Food, alcohol, cigarettes, E-cigarettes, Cannabis (hashish/marijuana), Cocaine, Ecstasy/MDMA, Psychedelics (magic mushrooms/truffles), Other.

- What are you doing right now?

With the following answer options: Eating, Working/Studying, Physical activity, Household tasks, Resting, Using social media, Watching a movie/series, Hobbies, Hanging out with friends, Personal care, On my way to somewhere, Something else.

- How much do you enjoy what you’re doing right now?
- How many people are you with right now?

With the options: 0, 1, 2, 3, 4, 5, 6 or more.

If this question is answered with anything other than “0” the following two questions are triggered:

- Who are you with? (You can select more than one answer.)

With the following answer options: Family, Partner, Friend(s), Colleague(s)/Classmate(s), Stranger(s).

- How much do you enjoy their company?
- *Please indicate what you ate since the last beep. (You can select more than one answer.)

With the following answer options: Nothing, Healthy snack, Unhealthy snack, Healthy meal, Unhealthy meal.

If this question is answered with anything other than “Nothing” the following question is triggered:

- Did you experience a loss of control while eating?
- *Did you smoke since the last beep?

With the options “Yes” and “No”. This question is only triggered if the participant states at baseline that he or she smokes.

Please indicate to what extent you agree with the following statements:

- **Since the last beep I felt like I was in control
- **Since the last beep I was able to concentrate
- **Since the last beep I have been worrying
- **Since the last beep I like how people are treating me
- **Since the last beep I did or said something without thinking first

Appendix B2 Daily items.

*Morning items, asked in the first beep of the day together with the momentary items:*

*Note*. Items in the momentary survey with an asterisk (*) are phrased “since the last beep” in the momentary survey, but “since you woke up” in the morning survey. Items in the momentary survey with an asterisk (**) are phrased “Since the last beep” in the momentary survey, but “Since I woke up” in the morning survey.

- Did you have a nightmare last night?

With the options “Yes” and “No”.

If this question is answered with “Yes” the following question is triggered:

- How distressing was the nightmare?

Answered in a vertical 7-point likert scale with the following options going from top to bottom: Not distressing at all, 2, 3, 4, 5, 6, Extremely distressing.

- How satisfied are you with last night's sleep?

Answered in a vertical 7-point likert scale with the following options going from top to bottom: Not satisfied at all, 2, 3, 4, 5, 6, Extremely satisfied.

- How rested do you feel?

Answered in a vertical 7-point likert scale with the following options going from top to bottom: Not rested at all, 2, 3, 4, 5, 6, Extremely rested.

- Did you use any of the following substances yesterday?

With the following answer options: Alcohol, Cigarettes, E-cigarettes, Cannabis (hashish/marijuana), Cocaine, Ecstasy/MDMA, Psychedelics (magic mushrooms/truffles), Other drugs; No, I did not use any of these substances.

*Evening items, asked at a fixed time late in the evening separately from the momentary items:*

Please indicate to what extent you agree with the following statements:

- Today I felt like I could count on my friends and/or family for support.
- I was able to handle today's challenges.
- Today, did you avoid any of the following? (You can select more than one answer.)

With the following answer options: Unpleasant interactions with somebody, Certain places or situations, Negative or hurtful thoughts, Daily activities, Scary or stressful objects or animals, Scary or stressful places, Scary or stressful activities, Pain-inducing activities, Unpleasant memories, Physical intimacy, No, I did not avoid any of these​.

For each of the selected options a question was triggered that looked like this:

- To what extent did you avoid [selected option] today?

Indicate if you engaged in any of the following activities today and, if so, to what extent

- I intentionally hurt myself
- I watched porn
- I checked my body
- I was obsessively thinking
- I felt the compulsions to do certain things
- I looked for information on the internet regarding my health
- I had a conflict
- I vomited on purpose
- I used laxatives
- In general how was your day?

Answered in a vertical 7-point likert scale with the following options going from top to bottom: Not good at all, 2, 3, 4, 5, 6, Extremely good.

- Today my sexual desire/drive was…

Answered in a vertical 7-point likert scale with the following options going from top to bottom: Not strong at all, 2, 3, 4, 5, 6, Extremely strong.

- Did you take your medication today?

With the options “Yes” and “No”. This question is only triggered if the participant states at baseline that he or she is taking medication for a mental health problem.

- Did you visit a healthcare professional today?

With the options “Yes” and “No”.

Appendix B3 Weekly items.

Triggered every 7 days at a fixed time.

Please answer the following questions thinking about the last 7 days:

- How many times did you engage in any sexual behavior? (alone or with someone)

With the following answer options: 0, 1, 2, 3, 4, 5, 6+.

- How satisfied are you with your sex life?
- Did you wish to die or disappear during this week?

Answered in a vertical 7-point likert scale with the following options going from top to bottom: Not at all, 2, 3, 4, 5, 6, A lot.

- Does your life have a clear meaning?
